# Supplementary material for: A Novel Chitosan Composite Biomaterial with Drug Eluting Capacity for Maxillary Bone Regeneration
Source: Materials (Basel). 2023 Jan 10;16(2):685. doi: 10.3390/ma16020685 (PMC9866710; doi:10.3390/ma16020685)
Supplement: Supplementary file 1 [file materials-16-00685-s001.zip › materials-2119647-supplementary.pdf]

Supplementary files.

Table S1. Solution molarities. Visible stability and manipulation of CS fibers at different NaOH solution molarities (0,05-0,5). The parameters were scored as low/bad, correct, or optimal marked with an X.

NaOH solution (M)	Low/bad	Correct	Optimal
0,05	X		
0,1	X		
0.5			X

Table S2: Needle diameters. Visible stability and manipulation of CS fibers manufactured with different needle diameters: 0,5 mm and 0,9 mm. The parameters were scored as low/bad = 0, correct = + or optimal = ++.

Needle diameter (mm)	Score
0,5	0
0,9	++

Table S3. Rate. Visible stability and manipulation of fibers at different CS percentages (1, 2 and 3%) and at different rates (60, 75, 100 and 150 mL/h). The parameters were scored as low/bad = 0, correct = + or optimal = ++.

	Rate (mL/h)			
CS (%)	60	75	100	150
1	0	++	++	++
2	+	++	++	++
3	0	++	++	++
